# SIGIRR: An Orphan Receptor Mediating Anti-inflammatory Actions

**DOI:** 10.1017/erm.2025.10009

**Published:** 2025-06-30

**Authors:** Siqi Cheng, Lingyue Cui, Jingyi Chen, Qianwei Xiu, Rujia Si, Xiaoguang Yang, Ying Shi

**Affiliations:** 1 Department of Hepatology, First Hospital of Jilin University, Changchun, Jilin, P.R. China; 2 Clinical Medical College of Jilin University, Changchun, Jilin, P.R. China; 3 National Engineering Laboratory for Druggable Gene and Protein Screening, Northeast Normal University, Changchun, Jilin, P.R. China; 4 The Affiliated Cancer Hospital of Nanjing Medical University, Jiangsu Cancer Hospital and Jiangsu Institute of Cancer Research, Nanjing China

**Keywords:** anti-inflammatory, IL-1R, interleukin-1, SIGIRR, TLRs

## Abstract

SIGIRR, also known as the single immunoglobulin interleukin-1 receptor (IL-1R)-related molecule, is a member of the IL-1 receptor superfamily and is believed to play a pivotal role in inflammation and anti-inflammatory regulation within the body. Studies have shown that SIGIRR expression is associated with autoimmunity, inflammatory disorders, graft rejection, viral infection, thrombosis and tumour progression. Due to its unique structure and function, SIGIRR is commonly referred to as an ‘orphan receptor’, with IL-37 being the only confirmed ligand molecule for SIGIRR to date. The primary mechanism through which SIGIRR exerts its anti-inflammatory regulatory effect involves the negative modulation of the Toll-like receptor-IL-1R (TLR-IL-1R) signalling pathway. TLR-IL-1R signalling plays critical roles in immune responses triggered by microbial invasion and alterations in the tumour immune microenvironment. This article provides an overview of research findings on SIGIRR as an orphan receptor and its regulatory role in maintaining a delicate balance between natural immune activation and uncontrolled inflammatory processes under pathological conditions.

## Introduction

SIGIRR, short for single immunoglobulin interleukin (IL)-1-related receptor and known as IL-1 receptor 8 (IL-1R8), is a protein belonging to the Toll-IL-1R (TIR) signalling receptor superfamily. It was first discovered and named by Thomassen et al. in 1999. SIGIRR has a unique structure consisting of a single Ig extracellular domain, a transmembrane domain, a conserved TIR intracellular domain and an intracellular tail region (Ref. 1). Although it was initially classified as part of the IL-1 receptor family, its structure differs from that of other members of the same family. The IL-1 receptor family consists of 10 members named IL-1R1 through IL-1R10 (Ref. 2). Compared with IL-1Rs, SIGIRR contains only one Ig extracellular domain, whereas IL-1Rs have three (Refs. 1, 2). Additionally, the TIR domain of SIGIRR lacks the essential amino acids Ser447 and Tyr536 required for signal transmission but instead relies on Cys222 and Leu305, which are crucial for maintaining its function (Refs. 3, 4).

The SIGIRR gene is expressed mainly in epithelial tissues, such as lymphoid organs, the liver and the gastrointestinal tract. Upon lipopolysaccharide (LPS) induction, the expression of the SIGIRR gene is downregulated in intestinal epithelial cells (IECs), bladder epithelial cells and hepatic parenchymal cells (Refs. 4–10). SIGIRR is highly expressed in leukocytes and can be found in dendritic cells (DCs), natural killer (NK) cells, T lymphocytes and platelets (Refs. 11–14). in vitro studies have shown that SIGIRR is highly expressed in epithelial cell lines such as HT29 and T84 cells but moderately expressed in DCs (Refs. 15, 16). Additionally, research by Xu et al. has shown that the expression level of human SIGIRR gradually decreases with age. This finding may be related to immune dysfunction and chronic inflammation (Ref. 17). Overall, SIGIRR has different expression patterns in various types of tissues and organs but plays an important role in immune regulation. SIGIRR is an orphan receptor that plays a crucial role in immune regulation. To date, IL-37 remains the sole confirmed cytokine ligand that interacts with SIGIRR (Refs. 4, 12). While prior studies have demonstrated a complex interaction network involving IL-37, SIGIRR and IL-18Rα, and sequence homology exists between IL-37 and IL-18, limited evidence supporting direct binding of IL-18 to SIGIRR has been established. (Refs. 18, 19).

Research shows that IL-37, as a ligand of SIGIRR, exists in two forms: cytoplasmic expression type and secreted expression type. Among them, cytoplasmic expression type IL-37b can form a complex with Smad3 and upregulate the expression of non-receptor protein tyrosine phosphatase through nuclear translocation, thereby promoting dephosphorylation and inhibiting the activation of tyrosine phosphorylation-dependent signalling pathways, including ERK, p38 MAPK, JNK, PI3K, NF-κB and STAT3 pathways (Ref. 20). Secreted IL-37, on the other hand, exerts its anti-inflammatory activity depending on SIGIRR. Under LPS stimulation, IL-37 can form a ternary complex with the α subunit of the IL-18 receptor (IL-18Rα) and SIGIRR and is assembled on the surface of peripheral blood mononuclear cells. The absence of IL-18Rα leads to a significant decrease in the anti-inflammatory activity of IL-37 (Ref. 21). In colonic organoid inflammation models and mouse skin cancer diseases, gene knockout of SIGIRR deprives IL-37 of its inhibitory effect on immune cell activation (Refs. 22, 23). In experimental autoimmune encephalomyelitis (EAE) mouse models, IL-37 prevents neurofunctional deficits and myelin loss through SIGIRR but has no effect on SIGIRR-deficient mice (Ref. 24). Recent studies have shown that delivery of the IL-37b gene via AAV9 vector can activate the SIGIRR pathway and prevent recurrent herpetic stromal keratitis in mice, and this pathway exhibits similar characteristics to human colorectal cancer (Ref. 25). Additionally, in cardiovascular diseases, the IL-37/SIGIRR axis can weaken platelet activation and thrombosis, suggesting its potential value as an antiplatelet therapy to reduce the risk of cardiovascular events (Ref. 26). This discovery provides a new research direction in the field of cardiovascular health and may promote the development of related drugs. Notably, the dysfunction of the IL-37/SIGIRR axis has been observed in HIV-infected individuals (Ref. 27). These data indicate that the positive expression of SIGIRR is necessary for the function of secreted IL-37. However, recent studies have shown that SIGIRR can also act independently of IL-37 as a blocker of Toll-like receptors (TLRs) and IL-1β signalling pathways in inflammation-related tissue injury diseases, mainly focussing on endothelial cells and liver parenchymal cells (Refs. 28, 29). The above SIGIRR-related anti-inflammatory responses are almost all achieved by inhibiting the downstream myeloid differentiation primary response 88 (MyD88)/IRAK/NF-κB signalling pathway.

## SIGIRR transcription

The regulatory mechanism of the SIGIRR gene transcription is not fully understood. Research has shown that upon substance P-induced stimulation, (TGF-β1) induces SIGIRR expression in M1 macrophages, and silencing p38 MAPK or TAK-1 can partially reverse this effect. In addition, Transforming growth factor β (TGF-β) is necessary for substance P-induced SIGIRR expression (Ref. 30). However, some contradictory views have been reported: on the one hand, certain studies suggest that silencing Sp1 upregulates the expression of SIGIRR in macrophages (Ref. 31); on the other hand, Sp1 is necessary for SIGIRR gene transcription in the HL-60 epithelial cell line cultured in vitro. LPS induction decreases the level of SIGIRR, and this decrease depends on Sp1 inhibition and p38 MAPK activation. Moreover, Sp1 has been shown to mediate SIGIRR transcription in IECs, and the inhibitory effects of tumour necrosis factor-alpha (TNF-α) and LPS may be related to a reduction in Sp1 binding to DNA sequences (Ref. 30). These findings suggest that different molecular mechanisms may exist to regulate SIGIRR gene transcription in immune cells and epithelial cells. Microglia are the predominant glial cells in the central nervous system (CNS) that express TLR2 and SIGIRR. β-amyloid peptide (Aβ) significantly suppresses SIGIRR expression in microglia while upregulating TLR2 expression. Anti-TLR2 antibody intervention effectively attenuates Aβ-induced inflammation and ameliorates hippocampal long-term potentiation deficits, concomitant with a marked increase in SIGIRR expression. Further investigations reveal that SIGIRR expression may be regulated via the Phosphatidylinositide 3-kinases (PI3K)/Protein Kinase B (AKT)/peroxisome proliferator-activated receptor (PPAR)γ signalling pathway, indicating that SIGIRR could serve as a critical target gene of the PPARγ pathway (Ref. 32). Other studies have proposed that resveratrol can induce SIGIRR expression by enhancing promoter transcriptional activity in hepatic parenchymal cells, suggesting that this pathway may be one important mechanism underlying its anti-inflammatory activity (Ref. 33).

## Signal transducers regulated by SIGIRR

As a transmembrane receptor, SIGIRR is most notably known for its ability to inhibit the signalling pathways of IL-1Rs and TLRs, which are also transmembrane receptors. These receptors, including IL-1R1, IL-1R5, ST2, TLR1–4, TLR7 and TLR9, interact with pathogen-associated molecular patterns (PAMPs) to transmit extracellular signals into the cell (Refs. 1, 4, 34). PAMPs consist of various biomolecules, such as LPS on bacterial surfaces, flagella and double-stranded or single-stranded DNA and RNA found in lysosomes or in the cytoplasm due to viral invasion. They can activate classical inflammatory pathways involving NF-κB and JNK. Research has shown that when SIGIRR forms complexes with these receptor/ligand complexes, it inhibits the dimerisation of MyD88, preventing the transduction of inflammatory signals (Refs. 20, 35). The immunoregulatory response mediated by TLRs in human airway epithelial cells (HAECs) is believed to be suppressed by SIGIRR. In HAECs treated with LPS, flagellin protein and CpG DNA, ectopic expression of SIGIRR leads to a reduction in the transcript levels of the inflammatory mediators IL-6 and TNF-α. SIGIRR inhibits the interaction between MyD88 and TLRs, resulting in attenuated immune responses mediated by TLR4, TLR5 and TLR9. Therefore, SIGIRR is considered a potential therapeutic target for acute respiratory distress syndrome (ARDS) (Ref. 36). SIGIRR can also block the formation of TRAM homodimers (Ref. 37). Limited research suggests that SIGIRR can inhibit TLRs without the involvement of IL-37, such as its ability to inhibit caspase-8 activity during coronary artery damage in individuals with Kawasaki disease (KD) or its ability to improve mitochondrial metabolism in individuals with non-alcoholic fatty liver disease (Ref. 29).

**Figure 1.**
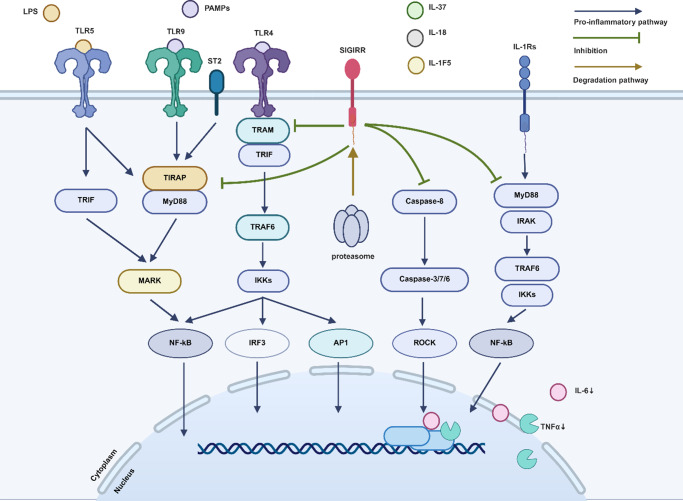
The regulatory signalling mechanism mediated by SIGIRR involves intricate interactions that modulate immune responses and inflammatory processes. SIGIRR is a transmembrane receptor, and its main function is to inhibit the IL-1Rs and TLRs signalling pathways. IL-1Rs and TLRs interact with PAMPs to activate the NF-κB and JNK inflammatory pathways. SIGIRR inhibits the dimerisation of MyD88 by forming complexes with these receptor/ligand complexes and weakens the immune responses mediated by TLR4, TLR5 and TLR9. Moreover, SIGIRR can block the formation of TRAM homodimers and prevent the transduction of inflammatory signals. In HAEC, the overexpression of SIGIRR can reduce the transcriptional levels of inflammatory mediators IL-6 and TNFα, and inhibit the activity of caspase-8 without involving IL-37. Under the induction of lipopolysaccharide, SIGIRR may be degraded through the proteasome pathway, thereby controlling and balancing the immune response in the body. *Source*: Created in BioRender. Qianwei Xiu.

IL-1R is evolutionarily conserved, with studies confirming the role of SIGIRR in maintaining homeostasis by reducing inappropriate inflammation amplification in species such as zebrafish, mice and pigs (Refs. 38, 39). Strong experimental evidence supports the negative regulatory function of SIGIRR in mice, with increasing clinical data confirming this function in humans. Shi et al. (Ref. 29) proposed that upon LPS induction, SIGIRR may undergo degradation via the proteasome pathway as part of the organism’s stress response to activate inflammatory pathways by downregulating conventional and IL-1R antagonistic receptors. Therefore, it holds significant clinical importance for controlling and balancing immune responses within the body (Figure 1).

## The involvement of SIGIRR in autoimmune disease

### Rheumatoid arthritis

Rheumatoid arthritis (RA) is a complex autoimmune disease in which the abnormal activation of memory CD4^+^ T cells plays a crucial role in its occurrence and progression. SIGIRR, a member of the IL-1 receptor family, acts as a negative regulator of the downstream signalling pathways of IL-1Rs and TLR, as well as the inflammatory response. The study by Liu et al. described the impact of SIGIRR on memory CD4^+^ T cells during the development of RA. The differential expression of SIGIRR on memory CD4^+^ T cells between healthy and RA cohorts was recorded. The data revealed a negative correlation between the percentage of memory CD4^+^ T cells expressing SIGIRR and disease activity in patients with RA. Mice lacking SIGIRR are more susceptible to antigen-induced arthritis, which is attributed to increased production of TNF-α by memory CD4^+^ T cells, whereas ectopic expression of SIGIRR leads to decreased production of TNF-α. Mechanistically, SIGIRR regulates the IL-1/EBPβ/TNF-α signalling axis. The deficiency of SIGIRR in memory CD4^+^ T cells during RA progression indicates that receptor induction can be targeted towards T cells, suggesting a potential new strategy for immunomodulatory therapy. In DCs, overexpression of SIGIRR inhibits TLR-induced cytokine production in macrophages and DCs, whereas SIGIRR knockdown leads to increased cytokine production after TLR stimulation. Additionally, overexpression of SIGIRR also suppresses the spontaneous release of cytokines in human rheumatoid arthritis synovial cells. in vivo experiments have revealed the role of SIGIRR as an anti-inflammatory agent, as SIGIRR (−/−) mice exhibited more severe disease in both zymosan-induced arthritis and collagen antibody-induced arthritis models (Ref. 13). Another study investigated the relationship between single-nucleotide polymorphisms (SNPs) and the mRNA expression profile of SIGIRR in the Chinese population and revealed that abnormalities in SIGIRR may be associated with the onset of RA, whereas the polymorphism rs7396562 of SIGIRR may be correlated with susceptibility to RA in the Chinese population (Ref. 40).

### Systemic lupus erythematosus

Researchers evaluated the functional role of SIGIRR in the pathogenesis of systemic lupus erythematosus (SLE), and the absence of SIGIRR is associated with increased activation of DCs and increased expression of various proinflammatory and antiapoptotic mediators. In the absence of SIGIRR, the number of CD4^+^ T cells is increased and the number of CD4^+^CD25^+^ T cells is decreased (Ref. 41). Additionally, the lack of SIGIRR increases B-cell activation and proliferation, including the production of autoantibodies against multiple nuclear lupus self-antigens. After peripheral blood mononuclear cells (PBMCs) from 48 patients with SLE and 38 healthy controls were evaluated using flow cytometry, the correlation between these cells and clinical data was analysed. The results revealed a significant increase in the percentage of Th17 cells in the peripheral blood of patients with SLE compared with healthy controls. Furthermore, in patients with active SLE, the percentage of Th17 cells was significantly higher than that in patients with inactive SLE. Compared with patients without lupus nephritis, patients with SLE also had a significantly increased frequency of Th17 cells and a decreased number of SIGIRR^+^ CD4^+^ T cells, along with an increased number of Th17 cells. These findings suggest that SIGIRR^+^ CD4^+^ T cells and Th17 cells may be involved in the pathogenesis of SLE (Ref. 42). Research has also revealed that SIGIRR levels in B cells are significantly higher than those in cells from healthy donors, which may lead to almost normal responses upon the stimulation of TLR7 or TLR9. Furthermore, while the number of IgG-secreting cells produced upon TLR9 stimulation decreases, both patients with SLE and healthy donors have similar abilities to induce B-cell differentiation into antibody-secreting cells through TLR signalling (Ref. 43).

In a drug-induced spontaneous SLE model, the induction of TLR-7 leads to the expression of type I interferon genes. As a member of the TLR family of suppressors, SIGIRR prevents SLE caused by hydrocarbon oil. The absence of SIGIRR results in the secretion of more IL-12 by DCs and increased expression of CD40 on the surface of splenic CD11C-positive DCs. A specific deficiency in SIGIRR increases the production of rheumatoid factors and anti-snRNP IgG, further exacerbating lupus nephritis in SIGIRR-deficient mice. SIGIRR inhibits TLR7-mediated DC activation through the interaction of its intracellular BB loop with TLR7 and MyD88, thereby preventing SLE induced by hydrocarbon oil use (Ref. 44).

SNPs were identified in patients with SLE in a Chinese population. A total of 741 patients with SLE and 731 healthy control subjects were enrolled in the study. The frequency of the T allele of rs7396562 in patients was significantly higher than that in controls. Some significant evidence for the association of the SIGIRR rs7396562 polymorphism with SLE was also observed in the dominant and recessive models. The associations of the SIGIRR rs7396562 T allele with clinical features were analysed, which revealed that photosensitivity and malar rash were significantly associated with the SNP (Ref. 45).

### Autoimmune encephalomyelitis

EAE and multiple sclerosis share similarities in terms of immune pathogenesis and lesions, both involving immune attacks on myelin basic protein in the CNS which lead to demyelination and various neurological impairments. The role of IL-37 in experimental autoimmune encephalomyelitis has been investigated, and recombinant human IL-37 has been confirmed to exert therapeutic effects on the EAE model. Its mechanism may involve reducing inflammatory responses through the IL-1R5/SIGIRR pathway, thereby protecting against neurofunctional deficits and myelin fibre loss in an experimental animal model (Ref. 24).

IL-17 is a proinflammatory cytokine that plays an important role in the host’s defence against infection, as well as in the development of various human and animal autoimmune diseases and the regulation of allergen-specific immune responses. Inhibiting IL-17 can significantly reduce the CNS production of chemokines and effectively alleviate EAE progression (Ref. 46). The study by Gulen et al. confirmed that SIGIRR plays a crucial role in negatively regulating IL-17 expression and that SIGIRR-deficient mice are more susceptible to EAE. Compared with wild-type T cells, SIGIRR-deficient T cells are more sensitive to IL-1 stimulation, leading to increased Th17 cell differentiation, suggesting that SIGIRR may influence Th17 cells by modulating IL-1 signalling during their differentiation. Compared with wild-type Th17 cells, SIGIRR-deficient Th17 cells exhibit increased IL-1-induced phosphorylation of c-Jun N-terminal kinase (JNK) and Mammalian target of rapamycin (mTOR) kinases, which inhibits the secretion of IL-17 (Ref. 47). These findings indicate that SIGIRR plays an important role in Th17 cell differentiation and proliferation, suggesting its significant regulatory role in the mechanism underlying the occurrence of IL-17-dependent EAE.

### Allergic rhinitis

Allergic rhinitis (AR) is a chronic inflammatory airway disease caused by abnormal T-cell reactions, and excessive inhibition of Th17 and Th2 cells is considered an effective treatment method for AR (Ref. 48). In 2018, Li et al. studied the potential role of IL-37/SIGIRR axis-mediated immune system regulation in patients with AR. A comparison of the expression levels of SIGIRR on resting and activated CD4^+^ T cells from patients with AR and healthy individuals revealed that both types of CD4-positive T cells from patients with AR presented significantly higher levels of SIGIRR expression than did the cells from the healthy control group, with the highest level observed in activated CD4-positive T cells from patients with AR. These data suggest that SIGIRR may be involved in the activation of CD4^+^ T cells during the pathogenesis of AR. Furthermore, SIGIRR may act as a negative feedback regulator of excessive Th17 and Th2 responses, playing an important role in inhibiting IL-17 and IL-4 production (Ref. 49).

### Asthma

Allergic asthma is a chronic inflammatory disease primarily associated with the activation of CD4^+^ helper T-cell type 2 (Th2) cells. Researchers widely believe that innate pattern recognition receptors play crucial roles in adaptive immune responses. Research has shown that during House Dust Mite (HDM) sensitisation/attack, the loss of the SIGIRR gene significantly reduces the expression levels of Th2 cell cytokines in mouse lungs and draining lymph nodes but has no effect on the expression levels of cytokines produced by Th1 or Th17 cells. SIGIRR-deficient mice exposed to HDMs exhibit reduced production of mucus-producing goblet cells and HDM-specific IgG1 antibodies and reduced airway hyperresponsiveness. Consistent with the weakened Th2 response, CCL24 expression for eosinophil chemotaxis and eosinophil counts around airways and bronchi are significantly decreased in SIGIRR-deficient mice. Conversely, the levels of IL-17A-responsive chemokines and neutrophil numbers are unaffected (Ref. 50). Another study by this team revealed that SIGIRR forms a complex with the IL-33 receptor ST2 and inhibits IL-33/ST2/NF-κB signalling in cultured cell lines. Compared with wild-type control mice, SIGIRR-deficient mice exhibit an enhanced IL-33-induced Th2 response, indicating that SIGIRR negatively regulates IL-33/ST2 signalling in vivo. This study described high SIGIRR expression in in vitro-polarised Th2 cells but not in Th1 cells, which explains why SIGIRR does not affect inflammatory cytokine expression in Th1 cells. SIGIRR-deficient Th2 cells produce increased levels of Th2 cytokines, including IL-5, IL-4 and IL-13. This study suggests another mechanism through which SIGIRR regulates the Th2 response in vivo, possibly by blocking ST2 function (Ref. 51).

However, a sequencing study of 24 patients with asthma identified 12 variants in the SIGIRR gene. Polymorphism and haplotype association tests were subsequently conducted using linkage disequilibrium mapping techniques on a cohort of 391 paediatric patients with asthma, 462 adult patients with asthma and 639 control subjects. These results suggest that allelic variants or haplotypes of the SIGIRR gene are not associated with the susceptibility to asthma or related phenotypes in the Japanese population (Ref. 52).

### Kawasaki disease

KD is an acute vasculitis that occurs in the paediatric population, and its progression can lead to coronary artery aneurysms, making it a major cause of heart disease in developed countries. The levels of IL-37 in the serum of patients with KD are significantly lower than those in healthy individuals, which is directly associated with decreased expression levels in venous endothelial cells. SIGIRR, a receptor for IL-37, plays a necessary role in protecting the coronary artery endothelium from injury (Ref. 53). A similar study revealed that the levels of SIGIRR are significantly reduced in serum samples from patients with KD and in endothelial cells treated with serum from patients with KD. Ectopic SIGIRR expression can significantly reduce the expression levels of apoptosis-related proteins such as TLR4 and cleaved caspase-8 induced by treatment with serum from patients with KD, suggesting that SIGIRR can inhibit endothelial cell apoptosis induced by serum from patients with KD. Based on previous understanding of the mechanism by which TLR4 affects caspase-8 activation, SIGIRR may reduce endothelial cell apoptosis and subsequent coronary artery damage by attenuating caspase-8 activity (Ref. 28).

## The involvement of SIGIRR in inflammatory disorders

### Intestinal diseases

Several studies have suggested that the expression of SIGIRR in IECs can play a dual role in defence and anti-inflammatory processes. Uncontrolled transcription of the SIGIRR gene may lead to the abnormal proliferation of IECs, resulting in excessive inflammation and antimicrobial responses that ultimately contribute to the development of intestinal diseases. The unique population of IECs directly interacts with bacterial biomass, and TLRs within this cell population precisely regulate signal transduction (Ref. 54). Rakof-Nahoum et al. (Ref. 55) demonstrated that aberrant activation of TLR signalling in IECs is associated with conditions such as coeliac disease, inflammatory bowel disease, colon cancer and transmissible colitis. Therefore, stable expression of SIGIRR as a negative regulator is crucial for maintaining signalling homeostasis within TLRs. In an experimental mouse model of colitis induced by dextran sulphate sodium (DSS), Liu et al. observed the impact of exogenous SIGIRR expression on the colonic mucosa. Compared with the control group, the congestion, oedema or erosion in the experimental group was significantly lower (Ref. 56). Yang et al. discovered that the absence of SIGIRR results in heightened inflammatory tension within the gastrointestinal tract. Compared with healthy controls, patients with ulcerative colitis (UC) present downregulated expression of SIGIRR in their colonic mucosa, indicating the potential involvement of SIGIRR in UC pathogenesis (Ref. 57). Additional studies have confirmed that mice lacking SIGIRR are more susceptible to developing colitis. In a DSS-induced colitis model, all SIGIRR knockout (SIGIRR^−/−^) mice died by day 15 of induction, whereas all wild-type mice survived. Compared with wild-type mice, a histological analysis of colon sections revealed more severe intestinal epithelial damage in SIGIRR^−/−^ mice, along with increased infiltration of inflammatory cells and elevated expression levels of the inflammatory cytokines IL-12, Interferon-gamma (IFN-γ), IL-17, IL-6 and IL-1β. Further investigations showed that SIGIRR^−/−^ mice developed colon tumours significantly earlier than their wild-type counterparts did and exhibited faster tumour proliferation rates, suggesting potential roles for SIGIRR in reducing colon cancer incidence and inhibiting tumour progression (Ref. 58). Ekaterina et al. (Ref. 59) found that necrotizing enterocolitis (NEC), a serious neonatal disease associated with various risk factors, including preterm birth and congenital heart defects (CHDs), is linked to specific genetic variants within the exons of the SIGIRR gene, as determined through Sanger sequencing analysis. These findings may have implications for assessing the risk of NEC development among full-term newborns with CHDs.

In conclusion, SIGIRR may be involved in regulating the defence mechanism of the intestinal epithelium, and its abnormal expression regulation is closely associated with the occurrence and development of intestinal diseases.

### Non-alcoholic steatohepatitis

Shi et al. reported a role for SIGIRR in the pathogenesis of non-alcoholic steatohepatitis. This study revealed the significant downregulation of SIGIRR expression in both patients with NASH and NASH mice compared with normal liver tissue. Genetic knockout of the SIGIRR gene exacerbated NASH progression induced by a high-fat and high-cholesterol diet, whereas transgenic overexpression of SIGIRR-ameliorated disease progression. Mechanistic investigations indicated that elevated levels of SIGIRR could counteract PPAR alpha (PPARα) suppression induced by free fatty acid (FFA) stimulation in hepatocytes, thereby reducing lipid-induced hepatocyte steatosis and attenuating sensitivity to LPS-mediated inflammatory factor release. Mass spectrometry data indicated that upon FFA stimulation, the decrease in SIGIRR expression in liver cells was partially attributed to the stress-induced upregulation of proteasome subunit beta type 20, leading to the nonspecific degradation of SIGIRR. These findings suggest that SIGIRR protects PPARα-regulated lipid metabolism and mitigates inflammatory responses triggered by external factors during NASH development, highlighting the potential use of SIGIRR agonists as therapeutic strategies (Ref. 29). Another study suggested that resveratrol can induce SIGIRR gene expression, thus alleviating Methionine-Choline Deficient (MCD)-induced NASH progression in mice (Ref. 33). The proteasomal degradation of SIGIRR has also been reported in previous studies of acute lung injury, indicating its presumed importance in the release of inflammatory signals.

### Renal fibrosis

In donor kidneys subjected to ischemia–reperfusion injury during kidney transplantation, phagocytes expressing both F4/80 and CD11c mediate proinflammatory responses and trigger adaptive immunity during transplantation through antigen presentation. However, after injury, these resident renal macrophages expressing the aforementioned surface markers transform into a reparative phenotype that is crucial for controlling inflammation and fibrosis. Cold ischemia and reversible ischemia–reperfusion injury suppress the antigen-presenting ability of renal macrophages, polarizing them towards the M2 phenotype. SIGIRR expression increases in these macrophages, reducing TLR4-mediated activation. Conversely, SIGIRR-deficient donor kidney macrophages tend to adopt an M1 phenotype and induce IFN-γ and IL-17 responses, leading to severe transplant fibrosis and abnormal humoral immune reactions. These findings suggest that SIGIRR plays a critical role in regulating the function of donor kidney macrophages after ischemia–reperfusion injury and has important implications for guiding their phenotypes and antigen presentation (Ref. 60). Additionally, another study presented some inconsistent evidence that under conditions of SIGIRR deficiency, the mRNA levels of proinflammatory or profibrotic mediators within the kidneys and their associations with morphological markers such as macrophage infiltration, T-cell numbers, tubular atrophy or interstitial fibrosis were not altered in a unilateral ureteral obstruction mouse model. These data suggest that molecules such as SIGIRR, TLR2, TLR9 and MyD88 may not play key roles in the mechanisms underlying obstructive renal fibrosis or tubular atrophy but may reveal their lack of susceptibility to renal fibrosis mechanisms (Ref. 61).

### Acute lung injury

The abnormal accumulation and subsequent excessive immune response of alveolar macrophages (AMs) play crucial roles in the pathogenesis of acute lung injury (ALI). Jiang et al. observed reduced SIGIRR expression in resident and recruited macrophages during ALI progression. This reduction was associated with concurrent increases in CD18 protein levels in LPS-challenged lung tissue. The overexpression of SIGIRR mitigated the recruitment of macrophages and neutrophils, decreased the production of inflammatory cytokines and ameliorated pulmonary pathology. Mechanistically, SIGIRR colocalised with CD18 in AMs and increased CD18 protein instability by promoting its ubiquitination and proteasomal degradation. A lack of CD18 counteracted the impact of SIGIRR overexpression on the efficacy of LPS-induced ALI treatment. These findings suggest that the synergistic interaction between SIGIRR and CD18 may play an important negative regulatory role in maintaining innate immune homeostasis in AMs during ALI pathogenesis (Ref. 62). HAECs may be involved in ALI and ARDS through a molecular mechanism mediated by TLRs. TLRs are present on the surface of HAECs, and interactions with their homologous ligands can trigger airway inflammation. One study examined the effects of different concentrations of LPS, flagellin protein and CpG DNA on the levels of homologous TLR4, 5 and 9 in the supernatant of HAECs overexpressing the SIGIRR molecule. The results showed that upon inflammatory stimulation, the overexpression of SIGIRR reduced the production of inflammatory mediators such as IL-6 and TNF-α. This attenuation was not due to decreased expression of TLR4, 5 or 9 but rather to the isolating effect of MyD88 on TLR signalling. Another study indicated that SIGIRR negatively regulates the ubiquitination of TRAF6. This inhibitory effect of SIGIRR increases the expression of TRAF6 in alveolar epithelial cells and AMs. SIGIRR knockdown may enhance the regulatory role of TRAF6 in Nuclear Factor kappa-B (NF-κB) activity through the classical rather than the nonclassical NF-κB signalling pathway. This regulatory mechanism between TRAF6 and SIGIRR can affect cytokine secretion and further exacerbate immune responses (Ref. 63).

### Organ transplantation

The TLR/IL-1R pathway mediates ischemia–reperfusion injury in transplanted organs and initiates immune responses. Studies have shown that SIGIRR expression is significantly increased in the transplanted kidneys of allogeneic mice and that knocking out SIGIRR triggers acute rejection reactions. Additionally, increased SIGIRR expression has been observed within the transplants themselves. Targeted deletion of SIGIRR has potent inhibitory effects on transplant tolerance and leads to the occurrence of acute rejection reactions. TLR4 and TNFα are highly activated in DCs from SIGIRR gene knockout receptor mice, resulting in increased IL-6 release. As a key factor regulating the renal alloimmune response, SIGIRR is considered a new target for controlling innate immunity and improving transplant survival rates (Ref. 64).

### Severe pancreatitis

The TLR4 signalling pathway plays a crucial role in the development of severe acute pancreatitis (SAP), and the associated pathogenic mechanisms are also important. Zhao et al. (Ref. 65) found that compared to wild-type RAW264.7 cells, RAW264.7 macrophages overexpressing SIGIRR presented significantly lower levels of TLR4, MyD88, IRAK-1 and TRAF-6 mRNA expression when cocultured with pancreatitis-associated ascitic fluid. Additionally, the levels of IL-2, IL-12, IL-17 and IFN-γ in the culture supernatant were significantly reduced, whereas the IL-10 level was increased. This study suggests that SIGIRR may be a promising target for treating SAP.

### Myasthenia gravis

Compared with healthy controls, the levels of IL-37 in the serum and PBMCs were significantly decreased in patients with myasthenia gravis (MG). Low levels of IL-37 were correlated with a more severe disease (quantitative MG score) and increased numbers of follicular Th (Tfh)/Tfh17 cells and B cells. Further research indicated that IL-37 plays a crucial role in regulating the immune response in patients with MG. By binding to its receptor SIGIRR, IL-37 can inhibit the activation of Tfh cells and B cells, reducing cytokine production. This inhibitory effect helps control the secretion of autoantibodies, thereby alleviating damage caused by abnormal immune function in patients with MG. Revealing the molecular basis for the immune imbalance during MG development and suggesting that IL-37 may be a novel target for treating MG and improving patient prognosis is important (Ref. 66).

### SIGIRR and tumorigenesis

NK cells are a type of innate lymphoid cell that possesses the ability to resist pathogens and plays an important role in activating and directing adaptive immune responses. Generally, NK cells play a secondary role in solid tumour progression (Ref. 67). Molgora et al. discovered SIGIRR as a checkpoint for regulating NK cell maturation and effector function. By genetically blocking SIGIRR, the resistance to NK cell-mediated hepatocellular carcinoma development, hematogenous liver and lung metastasis, and cytomegalovirus infection can be enhanced. In recent years, the roles of SIGIRR in the occurrence and development of various tumours have been gradually recognised. NK cells can combat viral infections and cancer. However, immunosuppressive cells present in the tumour microenvironment suppress the response of NK cells. Tumour progression leads to the recruitment and generation of regulatory T cells (Tregs) within the tumour, which are associated with a poor prognosis for cancer patients (Ref. 68). Sarhan et al. discovered that Tregs can inhibit the proliferation, IFN-γ production, degranulation and cytotoxicity of conventional rather than adaptive NK cells. Additionally, SIGIRR produced by Tregs contributes to phenotypic changes and the decreased function of conventional NK cells. Blocking PD-1, SIGIRR or IL-37 can eliminate the inhibitory effect of Tregs on conventional NK cells while maintaining the expression of TIM3 on NK cells. This study reveals a novel mechanism by which SIGIRR mediates the Treg-induced inhibition of conventional NK cells (Ref. 5).

## Role of SIGIRR in tumour immunity

### Lymphoma

In a mouse model of chronic lymphocytic leukaemia (CLL), the absence of SIGIRR exacerbated the progression of leukaemia, supporting its role as a novel tumour suppressor. This study aimed to detect the expression levels of the SIGIRR mRNA and protein in CLL cells and analyse their regulatory mechanisms. Compared with normal B lymphocytes, circulating leukaemia cells presented lower levels of SIGIRR expression (Ref. 69). Treatment with azacytidine restored the levels of SIGIRR in CLL cells, suggesting that DNA methylation may be involved in the regulation of SIGIRR expression and providing new therapeutic strategies. The loss of SIGIRR results in autoimmune reactions in lpr mice (a strain susceptible to lupus erythematosus), and sustained activation of NF-κB in the spleen increases the incidence rate of Diffuse Large B-Cell Lymphoma (DLBCL) in elderly mice. Treatment with nitrogen mustard can restore higher levels of SIGIRR expression in CLL cells, suggesting that DNA methylation may be involved in regulating SIGIRR expression (Refs. 69, 70).

### Renal cell carcinoma

The occurrence of renal cell carcinoma (RCC) is closely associated with inflammation, and the enrichment of IL-1 can promote the transformation of more tumour cells. In 12 samples from patients with clear cell RCC (ccRCC), the expression of the mRNA encoding the inhibitory receptor SIGIRR was consistently downregulated, which was consistent with the findings from in vitro-cultured tumour cell lines. ccRCC is a subtype in which SIGIRR is primarily downregulated, and its levels are also lower than those in normal and papillary or chromophobic tumour types. RNA sequencing analysis revealed that upon IL-1 stimulation, the ccRCC cell line A498 triggered the activation of inflammatory pathways and induced the expression of various genes that promote malignant transformation. However, the overexpression of SIGIRR in A498 cells can inhibit IL-1 signalling, suggesting that high levels of SIGIRR may have an inhibitory effect on RCC occurrence (Ref. 71).

### Prostate cancer

Research has shown that SIGIRR expression is associated with prostate cancer recurrence. SIGIRR is expressed mainly in the cytoplasm and nucleus of prostate epithelial cells, and no significant difference in the cytoplasmic expression of SIGIRR is observed between benign prostatic hyperplasia, high-grade prostatic intraepithelial neoplasia, prostate cancer and metastatic cancer. However, its expression is significantly reduced in the nuclear region of metastatic foci in prostate cancer. Results from a univariate analysis indicate that high cytoplasmic expression of SIGIRR is correlated with biochemical recurrence, whereas the results of the multivariate analysis suggest that in low-stage (pT2) tumours, SIGIRR can serve as an independent predictor of biochemical recurrence (Ref. 72).

### Precursor lesions of colorectal cancer

Research has shown that IL-37 plays an important role in the intestinal mutation, proliferation, apoptosis and migration of colorectal cancer (CRC) cells. IL-37 inhibits the protective cytotoxic T-cell-mediated immune response in the colitis-associated colorectal cancer and B16-OVA models. In IL-37tg mice, CD8^+^ T-cell function is impaired, characterised by reduced retention and activation, as well as a failure to proliferate and produce cytotoxic factors, allowing tumours to evade immune surveillance. IL-37 antagonises IL-18-induced CD8^+^ T-cell proliferation and effector function in an SIGIRR-dependent manner. The levels of IL-37 are significantly elevated in patients with CRC and are positively correlated with serum levels of the biomarker CarcinoEmbryonic Antigen (CEA) but negatively correlated with CD8^+^ T-cell infiltration in patients with CRC. These data suggest the potential use of IL-37/SIGIRR as a therapeutic target for CRC through the induction of cytotoxic T-cell deactivation (Ref. 73). Xiao et al. reported different findings, namely, that SIGIRR-deficient colonic epithelial cells exhibit a symbiotic bacteria-dependent steady-state defect and an increase in inflammatory gene expression, leading to an increased inflammatory response upon a DSS insult. Furthermore, in an acute otitis media + DSS-induced colitis model, the SIGIRR gene is specifically expressed in IECs in the context of SIGIRR deficiency, effectively reducing hypersensitivity reactions to DSS-induced colitis and tumour formation in SIGIRR^−/−^ mice (Ref. 56). The presence of a functionally inactive SIGIRRΔE8 gene has been observed in human colon cancer samples. The SIGIRRΔE8 transcript is generated through a selective splicing event that excludes the eighth exon of the SIGIRR gene. As a dominant-negative mutant, SIGIRRΔE8 captures the full-length SIGIRR protein within the endoplasmic reticulum (ER) by interacting with the ER-resident protein ribophorin I, preventing its glycosylation and membrane localisation. A comprehensive analysis of exome and RNA-sequencing data from 68 pairs of normal tissue and colorectal cancer samples revealed that the detected gene mutations were unrelated to the selective splicing of SIGIRRΔE8, highlighting the important role of splicing in epigenetic mechanisms (Ref. 74).

### Skin cancer

In the 12-Dimenthylbenz[a]anthracene (DMBA)/12-o-Tetradecanoylphorbol-13-acetate (TPA)-induced IL-37 transgenic mouse model, the inhibition of CD103-positive DC function exacerbated skin cancer development in mice. Mechanistically, SIGIRR suppressed the sustained activation of AKT, whereas IL-37 deprived DC cells of their resistance to tumour occurrence by regulating the SIGIRR-AMPK-Akt signalling axis, which is strongly associated with CD103-positive DCs. These findings suggest that IL-37 establishes an important connection between metabolism and the immune system through the regulation of CD103-positive DCs (Ref. 23).

### Breast cancer

Tumours adopt a series of strategies to alter their immune microenvironment and avoid clearance by immune cells, evading surveillance by the immune system. Studies have shown that SIGIRR is upregulated during breast epithelial cell transformation and in primary breast tumours and has an inhibitory effect on IL-1-dependent inflammatory pathway activation and cytokine production (Refs. 75–77). In these tumour microenvironments, the activation of NK cells is suppressed, while macrophages polarise towards the M2 phenotype. This state reduces the activation of NK cells and CD8^+^ T lymphocytes (effector T cells), thereby decreasing their ability to attack tumours. In mouse models of breast cancer lacking the SIGIRR gene, tumour growth and metastasis are significantly reduced. Further analysis revealed that after SIGIRR was knocked out, the activation of NK cells and CD8^+^ T lymphocytes was enhanced, leading to an increased attack on tumour tissue. These data suggest that high levels of SIGIRR expression may be an important factor contributing to immunosuppression, which is unfavourable for breast cancer treatment (Ref. 78).

### Tumour metastasis

NK cells are innate lymphoid cells that can resist pathogens and play important roles in activating and guiding adaptive immune responses. Researchers widely believe that NK cells play a secondary role in the progression of solid tumours. Molgora et al. discovered that SIGIRR acts as a checkpoint for regulating the maturation and effector function of NK cells. SIGIRR is upregulated during the differentiation of both mouse and human NK cells. A loss of SIGIRR leads to an increased frequency of mature NK cells in the blood, spleen, bone marrow and liver (Ref. 68). IL-18 is an important activating ligand for NK cells, and its sensitivity is regulated by SIGIRR on NF cells. Deletion of the SIGIRR gene results in increased expression levels of activation receptors on NK cells (including IFN-γ, granzyme B and Fas ligand). RNA-Seq and protein phosphorylation analyses demonstrated that, by modulating mTOR and JNK pathways activation, SIGIRR plays a role in hepatocellular carcinoma, sarcoma lung metastasis and CRC-induced liver metastasis models; thus, the loss of the SIGIRR gene can reduce tumour metastasis to the lungs and liver (Ref. 56, 74).

## Microbial infection

### HIV

Samarani et al. conducted a study on biological samples from different HIV-infected populations, and the results showed that untreated individuals with HIV infection presented decreased levels of cytokines in their cells, whereas treated individuals presented higher levels of cytokines. Long-term non-progressors presented higher serum concentrations of cytokines. SIGIRR expression in immune cells was downregulated in HIV-infected individuals; however, the serum level of its soluble form was increased. This trend was reversed in patients receiving antiretroviral therapy. Soluble SIGIRR may weaken the anti-inflammatory effects of cytokines. The serum levels of IL-37 and soluble SIGIRR are associated with certain clinical parameters in patients. Additionally, recombinant human IL-37 inhibits HIV replication in PHA cells (Ref. 27).

### HBV infection

Although antiviral drugs can effectively suppress the replication of hepatitis B virus (HBV), the maintenance of chronic inflammation in the liver is still considered a crucial factor contributing to the development of HBV-related liver fibrosis and advanced liver disease. Ye et al. (Ref. 7) showed that the overexpression of SIGIRR can inhibit the Hepatitis B X protein (HBX)-mediated activation of the MyD88/NF-κB inflammatory signalling pathway and the LPS-induced production of inflammatory cytokines induced in hepatocytes and HBV-replicating hepatocytes.

### Bacterial acute pyelonephritis

A study assessed the expression of SIGIRR in renal tissues during *Escherichia coli*-induced pyelonephritis and compared its role in the host immune defence before and after intervention with SIGIRR gene expression. The findings revealed predominantly positive SIGIRR protein expression in cortical tubular epithelial cells, whereas SIGIRR mRNA levels were significantly decreased in the pyelonephritis model. Additionally, a mouse urinary tract infection mouse model lacking the SIGIRR gene exhibited increased production of cytokines/chemokines and had a tissue bacterial load that was only 1/4200th that of control mice. This study suggested that SIGIRR hampers the effective host antibacterial immune response during the progression of pyelonephritis caused by uropathogenic *E. coli* (Refs. 79, 80).

### Platelet activation and thromboembolism

SIGIRR is expressed in different blood leukocytes, but its expression level is highest in human and mouse platelets. A lack of SIGIRR is associated with platelet hyperactivation under basal conditions, increased platelet aggregation after restimulation for thrombus formation and increased neutrophil–platelet aggregation induced by LPS and IL-18 in vitro. In an Denosine diphosphate (ADP)-induced pulmonary thromboembolism model, mice lacking SIGIRR are more susceptible to infection, primarily because of dysregulated IL-1 signalling (Ref. 14). Compared with healthy donor-derived platelets, platelets from patients with Systemic Inflammatory Response Syndrome (SIRS)/sepsis have decreased surface expression levels of SIGIRR, reflecting the disease severity (Ref. 81). Studies have shown that SIGIRR can be detected in released microvesicles or in plasma from septic patients, suggesting that its release through extracellular vesicles may contribute to reduced SIGIRR expression (Table 1).

**Table 1.** Pathophysiological roles of SIGIRR in disease

Pathological context	Disease	Role of SIGIRR	Selected Ref.
Autoimmunity	Rheumatoid arthritis	It affects the expression of memory CD4 T cells and inhibits cytokine release	(Ref. 13)
	Systemic lupus erythematosus (SLE)	Involved in the pathogenesis of SLE with CD4, T cells and Th17 cells; Interacts with Toll-like receptor (TLR)7 and myeloid differentiation primary response 88 (MyD88) to inhibit TLR7-mediated dendritic cell activation	(Refs. 41–44)
	Experimental autoimmune encephalomyelitis	The interleukin (IL)–1R5/SIGIRR pathway reduces inflammation and controls IL–17 expression and Th17 differentiation	(Ref. 47)
	Allergic rhinitis	Inhibit the production of IL–17 and IL–4	(Ref. 49)
	Asthma	Inhibition of IL–33/ST2/NF-κB signalling	(Ref. 51)
	Kawasaki disease (KD)	The apoptosis of endothelial cells induced by KD serum was inhibited	(Ref. 28)
Inflammatory disorders	Colitis	Regulate intestinal flora and maintain intestinal Toll-IL–1R signal homeostasis	(Refs. 54–58)
	Necrotising enterocolitis (NEC)	Specific genetic variations within the exon of the SIGIRR gene are associated with NEC	(Ref. 59)
	Non-alcoholic steatohepatitis	Protects peroxisome proliferator-activated receptor alpha-regulated lipid metabolism	(Ref. 29)
	Renal fibrosis	Regulate renal macrophage function	(Ref. 60)
	Acute lung injury	Negative regulation of innate immunity of CAMs and ubiquitination of TRAF6; Decreased production of IL–6 and TNF-α	(Refs. 62, 63)
	Severe pancreatitis (AP)	The cytokine expression of RAW264.7 macrophages was decreased	(Ref. 65)
Organ transplantation		Control the activation of TLR4 and TNF-α and affect the release of IL–6	(Ref. 64)
Myasthenia gravis		Binding with IL–37 inhibits the activation of T cells and B cells	(Ref. 66)
Cancer	Lymphoma	Control the expansion of monoclonal B cells	(Ref. 69)
	Renal cell carcinoma	Inhibit IL–1 signalling	(Ref. 71)
	Prostatic cancer	Predictors of biochemical recurrence	(Ref. 72)
	Precursor lesions of colorectal cancer	Induced cytotoxic T-cell inactivation	(Ref. 73)
	Skin cancer	Inhibit AKT signalling pathway	(Ref. 23)
	Breast cancer	Inhibition of IL–1-dependent inflammatory pathway activation and cytokine production	(Ref. 78)
	Tumorigenesis	Regulate the checkpoint of NK cell maturation and effector function and eliminate the inhibition of Tregs on conventional NK cells	(Refs. 56, 68)
Virus infection	Human immunodeficiency virus (HIV)	Weakens the anti-inflammatory effect of cytokines and inhibits HIV replication in PHA cells	(Ref. 27)
	Hepatitis B virus	Inhibition of MyD88/NF-κB inflammatory signalling pathway and production of inflammatory cytokines	(Ref. 7)
	Pyelonephritis	Hindering the host’s antimicrobial immune response	(Refs. 79, 80)
Thrombosis		Regulates IL–1 signalling and reflects SIRS/sepsis severity	(Refs. 14, 81)

## Conclusions

Considering the status of SIGIRR as an orphan receptor, transmembrane proteins with similar structures and functions to SIGIRR have not been confirmed. IL-37 is currently known to be a potential ligand for SIGIRR, whose ability to activate the occurrence and development of various diseases is weakened due to SIGIRR defects. Studies have also shown that, as a cell membrane receptor cofactor, SIGIRR is involved in regulating the signalling of other IL-1 superfamily ligands, such as IL-18. However, while SIGIRR is present in mice, the expression of the IL-37 gene is missing, and thus, whether SIGIRR represents a class of transmembrane protein molecules with special physiological functions remains unclear. Although SIGIRR is positively expressed in most cell types, its biological activity closely overlaps with that of TLRs and IL-1Rs. In general, its main role lies in anti-inflammatory regulation and further protection of tissue and organ functions by regulating microbial infections, lipid metabolism, tumorigenesis and ischemia–reperfusion injury during organ transplantation. Future research will focus on understanding the role of SIGIRR in specific cell types.

Some studies have suggested that mutations or genetic polymorphisms may affect the physiological function of SIGIRR. However, the detailed molecular mechanism involved remains unclear. This finding also implies that the localisation of SIGIRR may not be limited to the cell membrane since its deletion affects genes related to lipid metabolism, whereas previous reports suggest that this molecule may also migrate, similar to the migration of the TLR/MyD88 complex to mitochondria under specific physiological conditions.

Despite some conflicting findings reported in previous studies, overall, screening for agonists of this key inflammatory suppressor remains promising. For instance, inducing the expression of SIGIRR in CD4-positive T cells for the treatment of SLE, allergic rhinitis and asthma, or in endothelial cells for the management of KD and related intestinal disorders, or hepatocytes for hepatitis could potentially serve as a promising drug target to moderately inhibit the inappropriate inflammatory response.
